# Manual of Diseases of the Skin with an Analysis of 20,000 Consecutive Cases and Formulary

**Published:** 1898-12

**Authors:** 


MANUAL OF DISEASES OF THE SKIN with an Analysis of 20,000 Consecutive Cases and Formulary. By L. DUNCAN BULKLEY, A. M., M. D., Physician to the New York Skin and Cancer Hospital; Dermatologist to the Randall's Island Hospitals; Consulting Physician to the New York Hospital, Hospital for Ruptured and Crippled and Manhattan Eye and Ear Hospital, etc. Fourth edition, revised and enlarged. G. P. Putnam's Sons, New York and London. The Knickerbocker Press. 1898.

Any work which bears on its title page the name of Dr. L. Duncan Bulkley is certain to receive a cordial reception. The book now before us is rewritten, enlarged and practically entirely new, in every sense an advance upon the editions which preceded it. Owing to its conciseness as well as its simple and elementary character, it appeals most strongly to the student and busy practitioner as a ready work of reference.

Dr. Bulkley writes as a practical man, sensible and unpretentious, concentrating his energies upon the discussion of diagnosis and treatment and does so with an authoritiveness which his almost unique experience of an analysis of 20,oco consecutive cases fully justifies.

The chapters devoted to the anatomy and physiology of the skin, symptomatology and etiology are admirable, as being carefully presented and up to date. The classification adopted is one promulgated by the author and like that of Hebra's is based upon pathological anatomy. Its simplicity is commendable. The concluding chapter contains a general consideration of therapeutics, in which the remedies and methods of treatment are formulated, and systematised in a manner suitable for use against conditions of disease specially defined in the text.

